# Agri-Food Wastes as Natural Source of Bioactive Antioxidants: Fourth Edition

**DOI:** 10.3390/antiox15091175

**Published:** 2026-09-16

**Authors:** Silvana Hrelia, Maria Cristina Barbalace, Cristina Angeloni

**Affiliations:** Department for Life Quality Studies, Alma Mater Studiorum, University of Bologna, 47921 Rimini, Italy; silvana.hrelia@unibo.it (S.H.); cristina.angeloni@unibo.it (C.A.)

## 1. Introduction

The increasing production of agri-food waste represents one of the major challenges to the sustainability of modern food systems. Throughout the food supply chain—including harvesting, storage, processing, and distribution—large quantities of fruits, vegetables, and agricultural products are lost or discarded [1]. These residues, such as fruit peels, seeds, stems, leaves, and other underutilized plant tissues, are not merely waste materials but valuable sources of bioactive compounds [2]. Their valorization is therefore attracting increasing scientific and industrial interest as a strategy to reduce environmental impacts while creating new opportunities within the circular bioeconomy [3]. The fourth edition of the Special Issue “Agri-Food Wastes as Natural Source of Bioactive Antioxidants” aims to further explore the potential of these materials as sustainable sources of high-value bioactive compounds. Agri-food by-products are particularly rich in phenolic compounds, flavonoids, anthocyanins, carotenoids, terpenoids, vitamins, and other phytochemicals, many of which exhibit antioxidant, anti-inflammatory, antimicrobial, and cytoprotective properties [4,5]. Their recovery may therefore provide natural alternatives to synthetic compounds and support the development of innovative products for human health and well-being [6].

A key aspect of this research is the development of efficient and sustainable extraction methods. Although conventional solvent extraction remains widely employed, increasing attention is being directed toward green and innovative technologies, including ultrasound-assisted, microwave-assisted, pressurized liquid, pulsed electric field, and supercritical fluid extraction [7,8,9]. The use of safer and more environmentally compatible solvents, such as water, ethanol, ethanol–water mixtures, and natural deep eutectic solvents, is also particularly relevant [10,11]. The optimization of extraction processes should consider not only the recovery yield but also the preservation of bioactivity, selectivity, solvent and energy consumption, scalability, and economic feasibility. These aspects are essential for transferring promising laboratory approaches to industrial applications.

The bioactive compounds derived from agri-food wastes have considerable potential across several high-value sectors. In the pharmaceutical field, natural antioxidants and other phytochemicals may be promising candidates for developing new therapeutic or preventive strategies, particularly because oxidative stress and inflammation are involved in numerous chronic and degenerative diseases [12]. In the cosmetic and cosmeceutical sectors, waste-derived antioxidants may be incorporated into formulations aimed at protecting the skin from oxidative damage and environmental stress, while addressing the growing demand for natural and sustainable ingredients [13]. In the nutraceutical and functional food sectors, these compounds may be used as sources of natural antioxidants and other health-promoting molecules [14]. However, further investigations concerning bioavailability, stability, safety, mechanisms of action, and efficacy in appropriate biological models are necessary to support their effective application.

The valorization of agri-food waste is also strongly aligned with the United Nations 2030 Agenda for Sustainable Development [15]. In particular, it contributes to SDG 12 (Responsible Consumption and Production) by promoting waste reduction and resource efficiency, while supporting SDG 3 (Good Health and Well-Being) through the development of health-promoting products. It is also connected with SDG 9 (Industry, Innovation and Infrastructure) through the development of innovative green technologies and with SDG 13 (Climate Action) by reducing the environmental impact associated with waste generation and resource consumption.

This fourth Special Issue aims to bring together multidisciplinary contributions addressing the recovery, characterization, biological evaluation, and application of bioactive compounds from agri-food by-products. By integrating green extraction technologies with pharmaceutical, cosmetic, and nutraceutical applications, the valorization of agri-food waste can contribute to the transition toward more sustainable and circular production systems. Ultimately, transforming these residues into valuable bioactive ingredients represents not only an environmental opportunity but also a promising strategy for innovation, economic development, and the promotion of human health.

## 2. Overview of Published Articles

This Special Issue brings together two comprehensive reviews and ten original research articles that explore recent advances aimed at enhancing the functional potential and value of bioactive compounds, including phytochemicals, recovered from agri-food waste and by-products. Particular emphasis is placed on sustainable and green extraction strategies designed to maximize the recovery of valuable molecules while minimizing the use of hazardous organic solvents, energy consumption, and environmental impact. The contributions cover innovative approaches as well as the use of emerging green solvents, including water, ethanol, natural deep eutectic solvents (NADESs), and other bio-based solvent systems. These approaches represent promising alternatives to conventional extraction methods, offering improved extraction efficiency, selectivity, and sustainability while supporting the principles of the circular economy.

Overall, the studies collected in this Special Issue provide valuable insights into the recovery, characterization, and functional properties of food-waste-derived bioactive molecules, highlighting their potential as natural and sustainable alternatives for the prevention and management of health disorders frequently associated with oxidative stress.

The role of alternative solvents and the application of green extraction techniques have been extensively described in four research works.

Cranberry pomace is an underutilized source of valuable polyphenols and other bioactive compounds. The study of Otieno et al. (Contribution 1) demonstrates that solvent selection is crucial for maximizing the recovery of targeted antioxidants. Different ethanol-, methanol-, acetone-, formic acid-, and water-based mixtures produced markedly different phytochemical profiles and antioxidant activities. Among them, an acetone/methanol/water/formic acid mixture provided the most favorable extraction performance for phenolics and proanthocyanidins. These findings highlight the potential of tailored solvent systems within a green-economy framework, supporting more efficient valorization of agro-industrial by-products. Overall, optimizing solvent composition can improve extraction selectivity while promoting the sustainable recovery of high-value bioactive compounds from cranberry pomace.

Pistachio hulls are an abundant agro-industrial by-product and a potential source of valuable phenolic compounds. Their accumulation can create environmental concerns, making sustainable extraction and valorization strategies particularly relevant. The study of Paniagua-Garcia et al. (Contribution 2) optimized conventional and accelerated solvent extraction using response surface methodology. Accelerated solvent extraction achieved the highest phenolic recovery and antioxidant activity using 23% ethanol in water, highlighting the potential of aqueous ethanol as a more environmentally friendly solvent system. The extracts were particularly rich in gallic acid and other phenolic acids. Taken together, these findings support the green valorization of pistachio waste by combining efficient recovery of bioactive compounds with waste reduction and bioeconomy principles.

Apple pomace, a major by-product of juice and cider production, contains valuable fibers, vitamins, and polyphenols. Comparing hot-air convection and infrared drying, it has been demonstrated (Contribution 3) that infrared application reduced drying time and specific energy consumption while influencing polyphenol preservation. Măntăilă et al. also investigated ultrasound-assisted extraction as a sustainable strategy for recovering bioactive compounds. A natural deep eutectic solvent (NaDES) based on choline chloride and glycerol (1:1) achieved higher phenolic recovery and antioxidant activity than conventional solvents. The optimized extract was mainly characterized by flavan-3-ols, particularly catechin and epigallocatechin, followed by phenolic acids such as chlorogenic acid. Collectively, the findings highlight polyol-based NaDESs as promising green solvents for the sustainable valorization of apple pomace and the development of bioeconomy-oriented extraction processes.

Chestnut bur is an abundant agro-industrial waste rich in phenolic compounds with strong antioxidant potential. By optimizing accelerated and conventional solvent extraction to maximize total phenolic recovery, Paniagua-García et al. demonstrated (Contribution 4) that accelerated solvent extraction with 31.3% ethanol at 180 °C for 9 min yielded the highest phenolic content and antioxidant activity. The extract showed particularly high levels of gallic acid and 3,4-dihydroxybenzoic acid. The results identify aqueous ethanol as an effective and more sustainable solvent system for recovering valuable antioxidants from chestnut waste. Overall, accelerated solvent extraction offers a promising approach to chestnut bur valorization, contributing to green extraction, waste reduction, and circular-economy strategies.

Olive vegetation water (OVW), a by-product of olive oil production, is a rich source of phenolic compounds with antioxidant properties. The study of Mercatante et al. (Contribution 5) investigated OVW phenols as a natural alternative to partially or totally replace nitrites in cooked ham, a priority for both industries and consumers. During refrigerated storage, the extract effectively limited lipid and protein oxidation and reduced the formation of secondary oxidation products. Importantly, OVW phenols maintained sensory quality and enhanced color stability without negatively affecting consumer-relevant characteristics. Collectively, these findings demonstrate the potential of plant-derived phenolic extracts as cleaner alternatives to conventional preservatives in meat products, combining food-quality preservation with the sustainable valorization of olive oil by-products, supporting green processing and circular-economy principles.

Edible flowers also represent a promising natural source of phenolic compounds with antioxidant potential. Among the four species studied by Breda et al. (Contribution 6), Rosa de Santa Teresinha showed the highest phenolic content, while rose geranium and Rosa de Santa Teresinha exhibited the strongest antioxidant activity. The measured antioxidant capacity was far greater than that predicted from individual phenolic compounds alone. This suggests significant synergistic interactions among phenolics and other bioactive constituents. Multivariate analysis identified luteolin-7-O-glucoside and quercetin-3-O-galactoside as key contributors to antioxidant activity. Overall, these findings support the use of edible flowers as natural, multifunctional ingredients for sustainable nutraceutical and functional food applications.

By integrating green extraction technologies with the valorization of agri-food residues, the contributions further demonstrate how waste streams can be transformed into high-value sources of bioactive compounds with potential applications in nutraceutical, pharmaceutical, functional food, and other health-related sectors.

Four research papers focus on the identification of novel agri-food wastes as valuable sources of nutraceutical compounds. These studies highlight their potential to promote and protect human health through antioxidant, anti-inflammatory, and other bioactive properties.

The study of Peddio et al. (Contribution 7) explores purple-fleshed *Solanum tuberosum* cv. Vitelotte Noire peel as a sustainable source of nutraceutical compounds. The extracts were rich in phenolics, flavonoids, and anthocyanins, with alcoholic extracts showing the strongest antioxidant activity. All extracts also protected DNA from oxidative damage in vitro. Metabolomic analysis revealed a diverse profile of potentially bioactive metabolites, supporting the use of potato peel in the development of value-added functional foods and nutraceuticals.

The pericarp of mangosteen (*Garcinia mangostana* L.) was investigated as a valuable source of bioactive compounds with potential nutraceutical applications (Contribution 8). Three previously undescribed compounds, together with 18 known constituents, were isolated and evaluated for their anti-inflammatory activity. Several compounds markedly reduced nitric oxide production and modulated the levels of pro-inflammatory cytokines, including TNF-α, IL-6, and the anti-inflammatory cytokine IL-10. Among them, morusignin J emerged as a promising candidate, acting through the NF-κB and MAPK signaling pathways while enhancing markers associated with the anti-inflammatory M2 macrophage phenotype. Molecular docking further supported its interaction with inducible nitric oxide synthase (iNOS) and suggested favorable drug-like properties. Overall, the results indicate that mangosteen pericarp could represent a sustainable source of natural bioactive molecules for the development of antioxidant and anti-inflammatory nutraceutical ingredients.

Contribution 9 reports a study evaluating dragon fruit (*Hylocereus undatus*) peel powder as a sustainable functional ingredient for counteracting metabolic and hepatic alterations associated with excessive fat and fructose intake. The peel, naturally rich in dietary fiber, phenolic compounds, and betacyanins, was administered to rats fed a high-fat, high-fructose diet for 12 weeks. Supplementation significantly reduced body weight gain, visceral fat accumulation, insulin resistance, dyslipidemia, blood pressure, and systemic oxidative stress. In the liver, dragon fruit peel limited lipid accumulation and oxidative damage and modulated genes involved in lipid metabolism and inflammation. These findings highlight the potential of this agri-food by-product as a source of bioactive compounds for the prevention and management of metabolic and liver disorders.

Andrade et al. (Contribution 10) investigated coffee pulp, an abundant and bioactive-rich by-product of coffee production, as a potential dietary strategy for managing metabolic syndrome. A 10-week supplementation with coffee pulp improved several metabolic alterations induced by a high-fructose diet in rats, including excessive body weight gain, elevated blood pressure, hyperglycemia, and insulin resistance. At the hepatic level, it reduced lipid accumulation and oxidative damage while restoring glutathione levels. Coffee pulp also modulated the expression of genes involved in glucose and lipid metabolism in both liver and adipose tissue. Taken together, these findings suggest that coffee pulp could be valorized as a sustainable source of bioactive compounds with potential benefits for managing metabolic dysfunction and related health complications.

Finally, two narrative reviews examine, respectively, the potential application of condensed and hydrolysable tannins from agri-food waste in human and animal health and the use of agri-food waste products as a source of antioxidants for cosmeceutical application.

Camarda et al. (Contribution 10) describe agri-food by-products as sustainable sources of tannins with significant antioxidant potential and broad applications in human and animal health. Condensed tannins, particularly proanthocyanidins, act through free-radical scavenging, metal chelation, and activation of endogenous antioxidant defenses, supporting anti-inflammatory, metabolic, neuroprotective, and tissue-protective effects. Hydrolysable tannins also contribute to antioxidant protection while influencing antimicrobial activity and enzyme function. In animal health, these mechanisms may improve oxidative balance, immune function, tissue integrity, and feed efficiency. Overall, tannin-rich agri-food by-products represent promising natural antioxidant resources, although their biological effects depend on tannin structure, polymerization, and dosage.

Contribution 12 presents an overview of agri-food wastes used as sustainable sources of antioxidant bioactive compounds for high-value cosmeceutical applications. Importantly, this review provides an integrated perspective covering skin, oral, and hair care, highlighting the common molecular mechanisms through which these compounds exert their biological effects. Polyphenols, flavonoids, tannins, carotenoids, vitamins, lipids, peptides, and polysaccharides from peels, seeds, skins, pomaces, and other residues can counteract oxidative stress, modulate redox-sensitive pathways, reduce inflammation, and support tissue integrity. The review also discusses green extraction technologies that enable the sustainable recovery of these compounds. In summary, agri-food by-products represent promising resources for the development of innovative and sustainable antioxidant-based cosmeceutical products, although further studies are needed to ensure safety, stability, bioavailability, and clinical efficacy.

## 3. Conclusions

In conclusion, the valorization of agri-food waste should go beyond demonstrating that a by-product contains bioactive compounds. The real challenge lies in translating this knowledge into integrated and sustainable value chains in which these compounds can be efficiently recovered, characterized, validated, and transformed into high-value products. By combining waste reduction with green extraction technologies and the development of pharmaceutical, cosmeceutical, and nutraceutical applications, research in this field has the potential to contribute simultaneously to environmental sustainability, technological innovation, economic development, and human health. We hope that the contributions to this fourth edition will foster further research and collaboration toward a future in which agri-food waste is regarded not as an unavoidable burden, but as a valuable and renewable resource within a truly circular bioeconomy.
